# Pregnane × Receptor (PXR) expression in colorectal cancer cells restricts irinotecan chemosensitivity through enhanced SN-38 glucuronidation

**DOI:** 10.1186/1476-4598-9-46

**Published:** 2010-03-02

**Authors:** Caroline Raynal, Jean-Marc Pascussi, Géraldine Leguelinel, Cyril Breuker, Jovana Kantar, Benjamin Lallemant, Sylvain Poujol, Caroline Bonnans, Dominique Joubert, Frédéric Hollande, Serge Lumbroso, Jean-Paul Brouillet, Alexandre Evrard

**Affiliations:** 1 Institut de Génomique Fonctionnelle, Centre National de la Recherche Scientifique (CNRS) UMR5203, Institut National de la Santé et de la Recherche Médicale (INSERM) U661, Université Montpellier 1 et 2, Montpellier F-34094, France; 2 Laboratoire de Biochimie, Centre Hospitalier Universitaire, Nîmes, F-30029 France; 3 Laboratoire de Biochimie, Faculté de Médecine, Nîmes, F-30908 France; 4 Service d'Oto-rhino-laryngologie, CHU Nîmes, F-30029 France; 5 Service Pharmacie, Centre Régional de Lutte contre le Cancer Val d'Aurelle, Montpellier, F-34298 France

## Abstract

**Background:**

Clinical efficacy of chemotherapy in colorectal cancer is subjected to broad inter-individual variations leading to the inability to predict outcome and toxicity. The topoisomerase I inhibitor irinotecan (CPT-11) is worldwide approved for the treatment of metastatic colorectal cancer and undergoes extensive peripheral and tumoral metabolism. PXR is a xenoreceptor activated by many drugs and environmental compounds regulating the expression of drug metabolism and transport genes in detoxification organs such as liver and gastrointestinal tract. Considering the metabolic pathway of irinotecan and the tissue distribution of Pregnane × Receptor (PXR), we hypothesized that PXR could play a key role in colon cancer cell response to irinotecan.

**Results:**

PXR mRNA expression was quantified by RT-quantitative PCR in a panel of 14 colon tumor samples and their matched normal tissues. PXR expression was modulated in human colorectal cancer cells LS174T, SW480 and SW620 by transfection and siRNA strategies. Cellular response to irinotecan and its active metabolic SN38 was assessed by cell viability assays, HPLC metabolic profiles and mRNA quantification of PXR target genes. We showed that PXR was strongly expressed in colon tumor samples and displayed a great variability of expression. Expression of hPXR in human colorectal cancer cells led to a marked chemoresistance to the active metabolite SN38 correlated with PXR expression level. Metabolic profiles of SN38 showed a strong enhancement of SN38 glucuronidation to the inactive SN38G metabolite in PXR-expressing cells, correlated with an increase of UDPglucuronosyl transferases UGT1A1, UGT1A9 and UGT1A10 mRNAs. Inhibition of PXR expression by lentivirus-mediated shRNA, led to SN38 chemoresistance reversion concomitantly to a decrease of UGT1A1 expression and SN38 glucuronidation. Similarly, PXR mRNA expression levels correlated to UGT1A subfamily expression in human colon tumor biopsies.

**Conclusion:**

Our results demonstrate that tumoral metabolism of SN38 is affected by PXR and point to potential therapeutic significance of PXR quantification in the prediction of irinotecan response. Furthermore, our observations are pharmacologically relevant since many patients suffering from cancer diseases are often exposed to co-medications, food additives or herbal supplements able to activate PXR. A substantial part of the variability observed among patients might be caused by such interactions

## Introduction

Colorectal cancer is the fourth most common cancer in men and the third in women worldwide, and is currently undergoing a rapid increase in incidence [1]. Approximately two-thirds of patients present potentially curable disease but 30-40% will relapse with metastatic disease. Despite the emergence of targeted therapies, chemotherapy based on conventional fluoropyrimidine associated either with the platinum salt oxaliplatin or with the topoisomerase inhibitor irinotecan remains the first-line treatment [2]. Yet, clinical efficacy of these drugs is limited by the inability to predict chemotherapy outcome and toxicity. Notably, broad inter-individual variations in terms of response as well as of the occurrence of severe toxic side-effects like diarrhea and neutropenia are detected following treatment with compounds such as irinotecan [3]. In this context, identification of biological markers allowing the prediction of both therapeutic and toxic response is a priority issue.

Irinotecan (or CPT-11) is a water-soluble derivative of camptothecin acting as a topoisomerase I inhibitor and currently registered for use in patients with metastatic colorectal cancer. Irinotecan itself has weak, if any, pharmacological activity *in vitro*. It is thought to exert its antitumor activity *in vivo *after enzymatic cleavage by carboxylesterases 1 and 2 (predominantly in the liver but also partly at the tumor site) that generate the active metabolite SN38. Irinotecan and SN38 are then subjected to extensive intracellular catabolism yielding inactive metabolites. Irinotecan undergoes phase I oxidation by cytochromes P450 3A4 and 3A5 leading to oxidized inactive metabolites whereas SN38 is metabolised to SN38G through phase II glucuronidation by the UDP-glucuronosyl transferases 1A1, 1A6, 1A9 and 1A10 [4,5]. In addition, irinotecan and its metabolites are subjected to extracellular efflux through transporters, including P-glycoprotein (MDR1), multidrug resistance-related protein-2 (MRP2) and breast cancer resistance protein (BCRP) [6,7]. Numerous studies have focused on peripheral irinotecan metabolism, and genetic polymorphisms within genes coding for enzyme implicated in the irinotecan metabolic pathway have been extensively described. Notably, detection of the UGT1A1*28 genotype, found to be predictive for SN38 peripheral glucuronidation and irinotecan toxicity [8], is now recommended by the US Food and Drug Administration. However, conflicting results on UGT1A1*28 and the plethora of studies on others sequence variations in *UGT1A1*, but also in *ABCB1*, *ABCC1 *or *HNF1A *genes, suggests that reliable predictions of SN38 exposures cannot be based on the detection of a single polymorphism [9]. Inter-individual variation may be due to a combination of many genetic and non-genetic factors (diet, co-medications, *etc*.). Indeed, irinotecan pharmacokinetics and disposition is affected by various compounds now identified as ligands of the xenosensor PXR (Pregnane × Receptor, NR1I2) such as rifampicin [10] or St. John's wort [11].

PXR is a nuclear receptor acting as a "molecular sentinel" able to bind to a large variety of structurally diverse compounds included drugs, food additive or environmental toxics [12]. It coordinates the detoxification of many lipophilic xenobiotics *via *transcriptional regulation of a large number of metabolizing enzymes and transporters [13]. Targets genes of PXR are CYP3A4 [14], MDR1 [15], CYP2B6 [16], members of UGTs superfamily [17] and transporters like the multidrug resistance-related protein-3 (MRP3) [18] or the organic anion transporting polypeptide-2 (OATP2) [19]. PXR is predominantly expressed in liver and in intestinal tract, but little is known about its expression in tumors. Because PXR controls the expression of key genes involved in anticancer drugs disposition, recent works have focused on its potential role in drug resistance [20]. For instance, PXR is suspected to play a role in both all-trans retinoic acid [21] and etoposide [22] resistances through an enhancement of their CYP3A4-mediated metabolism. In addition, it has been shown that PXR induces cell proliferation and inhibits apoptosis in human colon cancer cells [23]. Considering the metabolic profile of irinotecan and the tissue distribution of PXR, we aimed to assess to what extent PXR could affect metabolism and colon cancer cell response to irinotecan. We show that expression of PXR in human colorectal cancer cells led to irinotecan and SN38 chemoresistance through enhancement of its glucuronidation.

## Materials and methods

### Cell lines, plasmids and transfections

The human colorectal cancer cells LS174T were kindly provided by Dr. Pierre Martineau (IRCM, Montpellier, France). SW480, SW620, HCT116, HT29, HepG2 and HuH7 were from the cells collection of the Macromolecular Biochemistry Research Center (Montpellier, France). All cell lines were grown in RPMI 1640 supplemented with 10% fetal calf serum (FCS), 2 mmol/l glutamine, 100 units/ml penicillin and streptomycin. Selection mediums for transfected cells were supplemented with 250 μg/ml (SW480 and SW620) or 500 μg/ml (LS174T) geneticin. Cells were maintained routinely at 37°C in 5% CO2 humidified atmosphere.

PXR expression vector was built by cloning hPXR-1 cDNA (NM_003889) [14] in a pcDNA3 vector (Invitrogen). Stable clones overexpressing PXR were obtained by transfecting cells with the pcDNA3-hPXR vector using lipofectamine LTX transfection reagent (Invitrogen), according to manufacturer's instructions. Parent LS174T cells were transfected with empty pcDNA3 vector to yield control mock-transfectant. The shRNA-expressing vectors were constructed by cloning shRNA expression cassettes into FG12 lentiviral vector [24] (additional file 1). Cells were transduced with lentiviral vectors and GFP positive cells were isolated using a BD FACSAria™ cell sorter as previously reported [25].

### Human specimen samples

Specimens of liver and colon biopsies were obtained from the pathologist after resection according to French government regulations and with approval of the ethical committee (Montpellier and Nîmes Hospitals). Informed consent was obtained from all patients. Tissue samples were stored in liquid nitrogen until further use.

### Chemicals

Irinotecan, 5-fluorouracil (5-FU), oxaliplatin and verapamil chlorhydrate solutions were provided by the department of Pharmacy of the Nimes university hospital. SN38 was a kind gift from Dr E. Chatelut (Claudius Regaud Institute, Toulouse, France). Dimethysulfoxide (DMSO), rifampicin, ketoconazole, fumitremorgin C and L-Sulforaphane (SFN) were purchased from Sigma-Aldrich.

### RNA extraction and reverse transcription

Total RNA were extracted using RNAeasy kit (Qiagen), according to the manufacturer's instructions. RNA quantity and quality of samples were determined by the 260:280 nm absorbance ratios using a NanoDrop spectrophotometer (Thermo Fisher Scientific). One μg of total RNA from each sample was added to 8.4 μl of reverse transcription mix containing 4 μl of first strand buffer 5×, 0.4 μl of dNTP mix 25 mM, 2 μl of dithiothreitol 10×, 1 μl of oligodT primer solution and of MLV-RT enzyme 200 U/μl. Solution volumes were adjusted to 20 μl by adding RNase free water. Samples were placed at 37°C for 1 hour and at 65°C for 5 minutes. cDNA solution volumes were adjusted to 100 μl by adding 80 μl of PCR grade water and stored at -20°C for further analysis.

### Real-time quantitative PCR

mRNAs expression was evaluated by RT-quantitative PCR, using a LightCycler 480 real-time PCR system and SYBRGreen PCR master mix 2× (Roche Diagnostics) in 96-well plates. Quantitative PCR was done using gene-specific primers and β-actin was used as reference gene (additional file 2). Standard curves were generated for all genes by serial dilution of cDNAs. After normalization of threshold cycle values with the amount of β-actin, gene expression levels were expressed as ratios compared with that of vehicle-treated cells. Each sample was run three times in duplicates, and data were analyzed using the 1.5 version of LightCycler 480 software (Roche Diagnostics). Standard curves were generated for all genes by serial dilution of cDNAs from LS174T control for relative quantification in cultured cells and from a pool of human liver biopsies for relative quantification in tumors.

### Western Immunoblotting

Protein extracts were prepared from cells by using M-PER^® ^mammalian protein extraction reagent (Thermo Scientific) in presence of a protease inhibitor cocktail (Roche), according to the manufacturer's protocol. Proteins (40 μg/lane) were separated by 12% SDS-polyacrylamide gel electrophoresis and transferred to a PROTRAN^® ^nitrocellulose membrane (Schleicher and Schuell). Membranes were sequentially incubated with anti-hPXR (G-11, Santa Cruz Biotechnology) or anti-βactin (Santa Cruz Biotechnology) primary antibody, and with peroxidase-conjugated anti-mouse IgG (Santa Cruz Biotechnology). Signals were detected by chemoluminescence using ECL Western Blotting Detection reagents (GE Healthcare).

### Immunohistochemistry

Tissues were embedded in paraffin and sections (5 μm) were dewaxed in a xylene bath and rehydrated in graded alcohols. Endogenous peroxidase activity was quenched with 1.5% H_2_O_2 _in methanol for 20 min. and washed in PBS. Antigen retrieval was performed by boiling slides in 10 mM sodium citrate buffer, pH 6.0. Nonspecific binding sites were blocked with 1% BSA, 3% normal goat serum, and 0.2% Triton X-100 in PBS for 1 h at RT. Slides were incubated with the primary anti-human polyclonal PXR antibody (Lifespan Biosiences) overnight at 4°C in 50 times-diluted blocking buffer. Universal immuno-peroxydase polymer anti-mouse Histofine^® ^(Nichirei Biosciences, Japan) was used as a secondary reagent, stainings were developed with DAB (brown precipitate, SIGMA) and hematoxylin counterstain was used. After dehydration, sections were mounted in Pertex (Histolab). Then, slides were scanned with high resolution Nanozoomer (Hamamatsu).

### Neutral red chemotherapeutic sensitivity assays

Experimental conditions for neutral red assays were adapted from a previously described protocol [26]. Briefly, 20.000 cells were seeded in 96-well microtiter plates. After 24 h incubation, cells were treated for 72 h with increasing concentrations of cytotoxics. After a neutral red incubation at 37°C for four hours, cells were washed with PBS and destained with 150 μl of 1% glacial acetic acid/50% ethanol (vol:vol). The absorbance at 540 nm was measured using a microplate reader (iEMS, Labsystems). The effect of the drugs on cell survival was expressed as the percentage of cell viability compared to untreated cells.

### Irinotecan metabolites detection assay

Cells were seeded in six-well plates at 10^6 ^cells/well, incubated for 24 hours, and then treated with 0.1% DMSO (solvent) or 10 μM SN38 for 24 h. Cell pellets and supernatants were stored at -80°C for further analysis. Cell pellets were dissolved in 500 μl of a mixture of methanol-acetonitrile (50:50 vol:vol). 400 μl of culture supernatants were added to 800 μl of the mixture of methanol-acetonitrile (50:50 vol:vol). After proteins denaturation by full-speed vortex mixing, samples were then centrifuged at 13000 rpm for 3 minutes. 550 μl of clear supernatants were mixed to 250 μl of 1 M HCl and used for HPLC injection. Irinotecan and its metabolites were detected and quantified by a HPLC method as previously described [27].

### Statistical analysis

The Mann and Whitney test was used to analyze the difference between two groups of quantitative variables. Alpha value was set at 5%. For comparisons among three groups of quantitative variables, the Kruskal Wallis test was used. In cases where there was a significant difference between the groups, a pairwise comparison was carried out by adjusting the alpha risk by the method of Bonferroni. Student's *t*-tests were performed when indicated in figures legends. All statistical analyses were carried out by the Department of biostatistics, epidemiology, public Health and medical information of the Nîmes University Hospital using the SAS software (SAS Institute Inc.).

## Results

### Expression of hPXR in colon tissues and colon cancer cells

**Figure 1:**
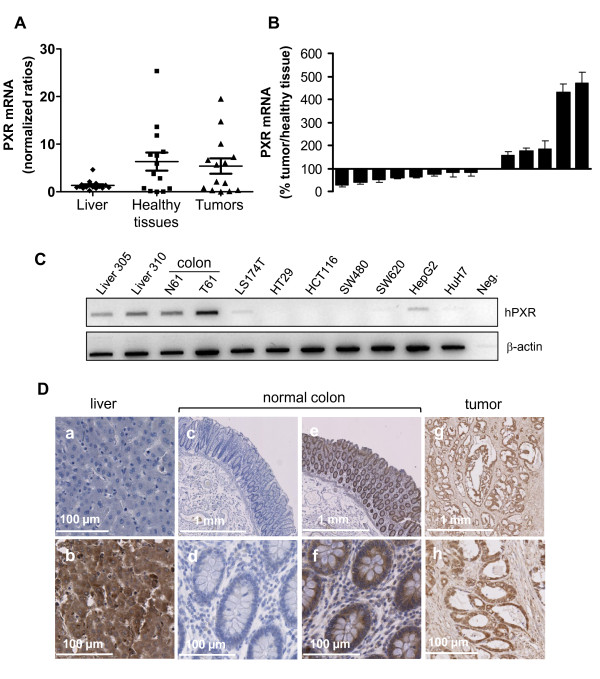
**Evaluation of PXR expression in human colorectal tissues and cell lines**. **A**, PXR mRNA expression measured by quantitative real-time PCR in human livers (n = 17) and colon samples (tumoral and healthy adjacent tissue, n = 14). Bars on graph represent mean PXR mRNA expression and SEM. **B**, Percentage of PXR expression in colon tumors compared to adjacent healthy tissue; bars, SEM. **C**, PXR mRNA expression in various colon (LS174T, HT29, HCT116, SW480, SW620) and hepatic (HepG2, HuH7) cell lines compared to livers (FT305 and 310) and to normal (N61) and tumoral (T61) colon tissues. **D**, Immunocytochemical staining for PXR on liver, normal colon and tumors tissues. Liver was used as positive control for PXR expression (b), photos a, c and d display negative controls without primary antibody.

To examine whether hPXR is expressed in human colon *in vivo*, we analyzed its mRNA expression in both normal and neoplastic human colon tissues. Human liver tissues, which are known to have high-PXR expression, were used as positives controls. As shown in figure 1A, PXR mRNA was detected in both normal and cancerous human colon tissues. In our panel of 14 patients, PXR mRNA was more abundant in some colon tissues than in liver biopsies, and displayed a greater variability in colon tissues than in liver tissues. Note that, although we did observe some quantitative differences between the expression of PXR in normal tissues and their matched colon tumour samples, we found no clear trend of tendency (figure 1B). In addition, we observed a very low expression of PXR mRNA in both colon cancer cells lines LS174T, SW480, SW620, or HT29 and hepatic cell lines HepG2 and HuH7 (figure 1C). This result on cell lines is in accordance with the previous observation that cultured cells often loose metabolic abilities and PXR expression [28]. Representative examples of PXR immunostaining are displayed in figure 1D, liver were used as positive control for PXR expression and negative controls without primary antibody are shown (photos a, c and d). We observed a strong immunostaining of PXR in both normal colon and tumors consistent with mRNA results.

### Functional characterization of PXR transfected LS174T colorectal cancer cells

**Figure 2:**
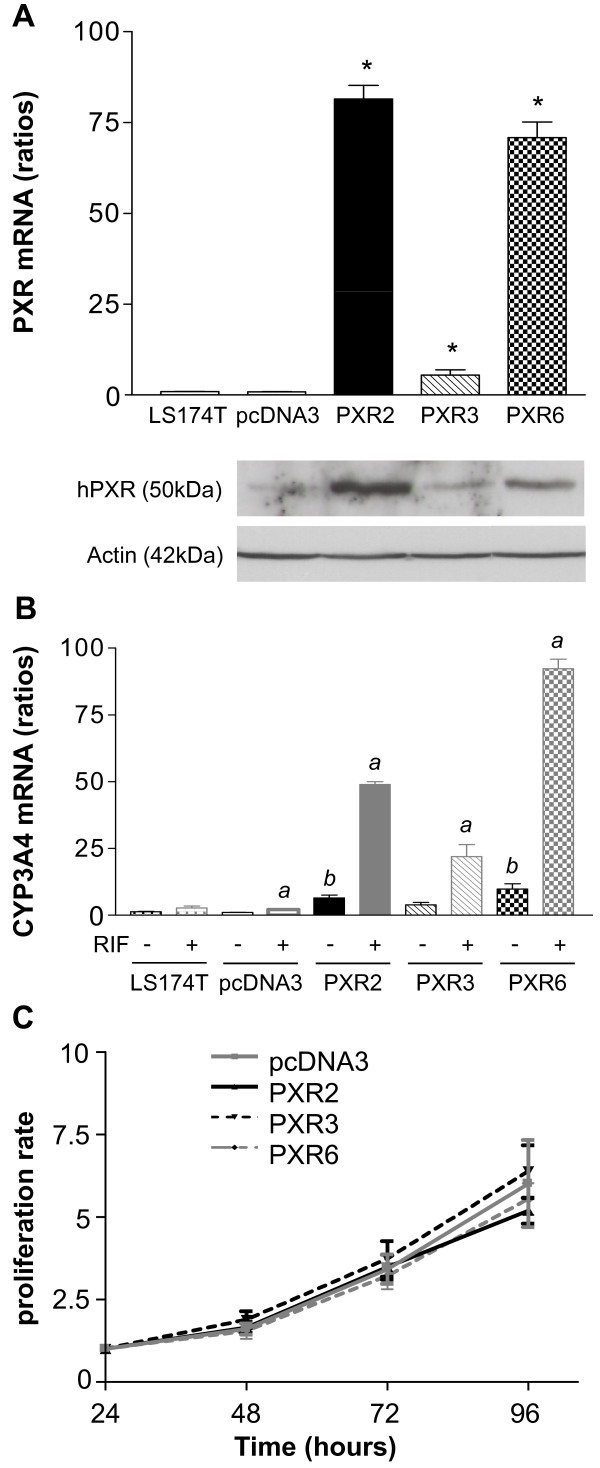
**Characterization of LS174T PXR-transfected cells**. **A**, PXR expression level in parent LS174T, pcDNA3-transfected and stable clones PXR2, PXR3 and PXR6 (top: mRNA expression level; bottom: protein expression level). * p < 0.05, PXR expression of stable clones compared to parent and pcDNA3-transfected cells, assayed by Mann and Whitney test. **B**, CYP3A4 mRNA expression levels of CTRL and PXR overexpressing clones treated 24 h by solvent (0.1% DMSO) or 10 μM rifampicin in serum-free medium. Results were obtained from three separate experiments; bars, SEM. *a*, CYP3A4 expression of cells treated by rifampicin compared to the vehicle treated groups, *b*, CYP3A4 expression of vehicle treated stable clones compared to vehicle treated; assayed by Mann and Whitney test (p < 0.05). **C**, proliferation rate of cell lines. Cells were seeded in six-well plates at 5 × 10^5^cells/well and counted at the indicated times after seeding (Z1 counter, Beckman Coulter). Data from three separate experiments; bars, SEM.

To decipher the role of PXR in irinotecan-mediated toxicity in colorectal cancer cells, we first established stable clones overexpressing hPXR in LS174T. These cells were chosen because of their low, but detectable, PXR mRNA expression, and their well known sensitiveness toward both irinotecan and SN38 [29]. In addition these cells express a functional p53 [30], which is partly involved in irinotecan response [31]. As shown in figure 2A, we selected three clones harbouring different PXR expression levels at both mRNA and protein levels (PXR2 ≈ PXR6 >> PXR3). Basal expression levels of CYP3A4 mRNA were higher (up to 9.31 ± 2.40 fold for PXR6) in PXR-expressing cells compared to controls (figure 2B) and were further increased by rifampicin (48.92 ± 1.07 fold for PXR2, 19.02 ± 6.83 fold for PXR3 and 92.25 ± 3.60 fold for PXR6 compared to controls). In agreement with the very low level of endogenous PXR detected in these cells, we observed a very weak increase of CYP3A4 mRNA levels upon treatment of parent and pcDNA3-transfected cells with rifampicin. The growth rate of PXR-transfected cells did not significantly vary from that of pcDNA3-transfected cells (figure 2C)

### PXR induces colorectal cancer cells resistance to irinotecan and SN38

**Figure 3:**
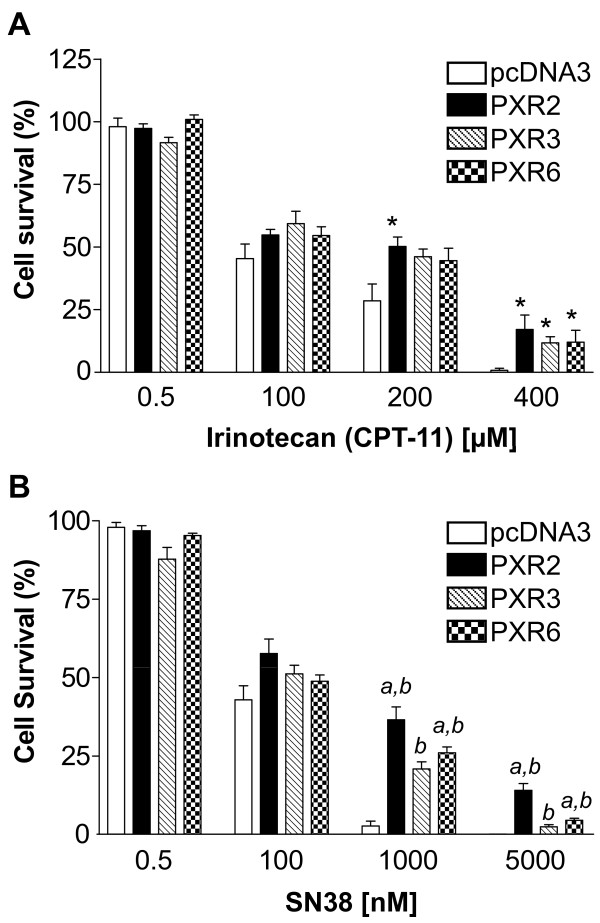
**Increased chemoresistance in PXR overexpressing cells to irinotecan and SN38**. For neutral red assays, cells were treated for 72 h by increasing concentrations of irinotecan (**A**) or SN38 (**B**). *Columns*, mean viability as a percentage of control (i.e., cells without chemotherapeutics treatment, 100%) from replicates (n = 6) from six separate experiments; bars, SEM. *a*, viability percentages of PXR2, PXR3 and PXR6 compared to pcDNA3-transfected cells (p < 0.05) (Mann and Whitney test). *b*, viability percentages of PXR2, PXR3 and PXR6 compared to each other using Kruskal Wallis test (p < 0.05).

To evaluate the role of PXR onto cell sensitivity to drugs used in the treatment of advanced colorectal cancer we first carried out neutral red cell viability assays. For this purpose, control and PXR-transfected LS174T clones were maintained for 72 hours in the presence of increasing concentrations of irinotecan, SN38, 5-fluorouracil or oxaliplatin. As shown in figure 3, PXR expression clearly led to an increased survival of LS174T cells towards irinotecan and SN38, and the level of chemoresistance to SN38 was correlated to the relative PXR expression level of each clone (figure 3B). Upon treatment with 1 μM SN38, PXR-transfected cells displayed a 6-to 9-fold higher viability (36.56% ± 4.05 surviving cells for PXR2 and 26.05% ± 1.82 for PXR6) compared to pcDNA3-transfected cells (3.93% ± 1.64). We found no difference in topoisomerase I activity between pcDNA3-transfected cells and PXR-transfected cells demonstrating that the observed chemoresistance is not due to a variation of topoisomerase I expression after PXR transfection (additional file 3)

As expected, cells were more sensitive to SN38 than to irinotecan, the latter undergoing minimal conversion into its active metabolite due to very low expression level of carboxylesterases in these cells (additional file 4). In addition, cell viability assays performed in SW480 and SW620 cell lines stably transfected with hPXR, showed similar PXR-dependent enhancement of SN38 resistance (additional file 5). On the other hand, we found no effect of PXR expression on cell sensitivity to both 5-fluorouracil and oxaliplatin sensitivities (additional file 6).

**Figure 4:**
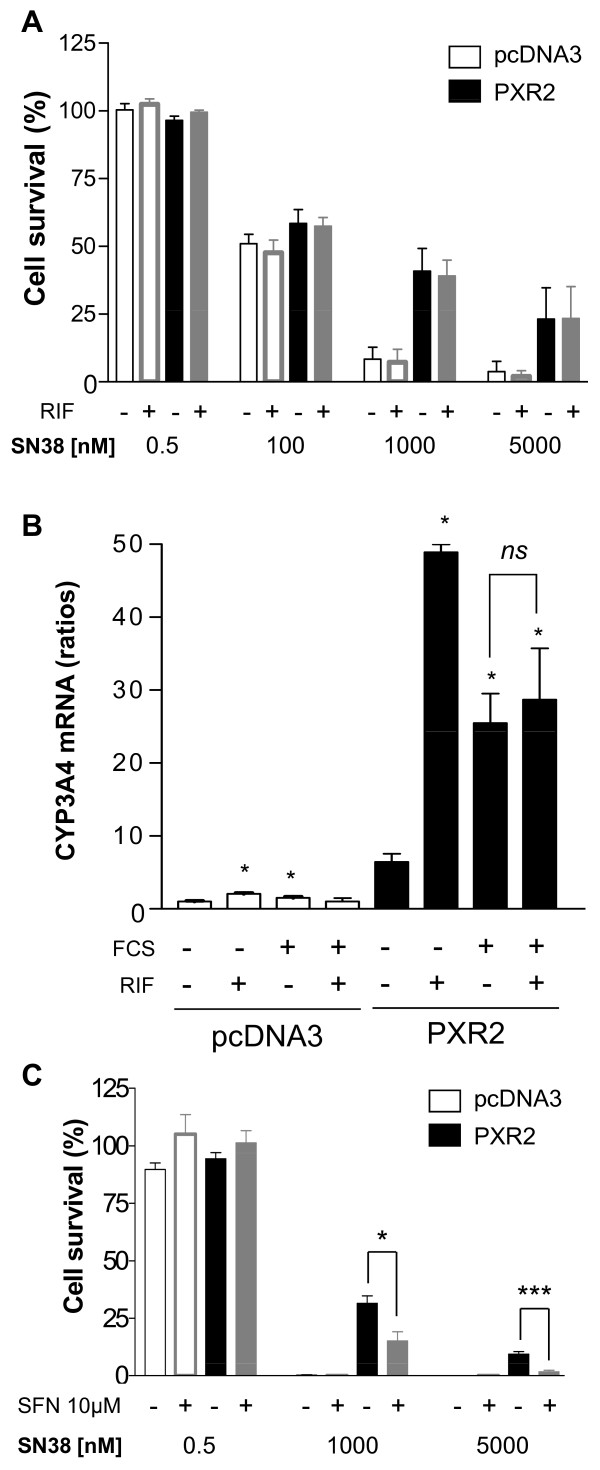
**A, cell viability assay on pcDNA3-transfected and PXR2 cells treated with rifampicin (RIF)**. Cells were cultured 24 h with DMSO 0.1% (solvent) or 10 μM rifampicin in 10% serum containing medium and then exposed to SN38 for 72 h. *Columns*, mean viability as a percentage of control (i.e., cells without chemotherapeutics treatment, 100%) from replicates (n = 6) from three separate experiments; bars, SEM. **B**, CYP3A4 mRNA expression level of pcDNA3-transfected or PXR-expressing cells cultured with (10%) or without serum and treated with solvent (0.1% DMSO) or 10 μM rifampicin for 24 h. Results were obtained from three separate experiments; bars, SEM. * p < 0.05 (Mann and Whitney test), CYP3A4 mRNA expression of cells cultured with serum, with or without rifampicin compared to vehicle treated cells. **C**, cell viability assay on pcDNA3-transfected and PXR2 cells treated with PXR antagonist L-Sulforaphane (SFN). Cells were cultured in presence of solvent (DMSO 0.1%) or 10 μM L-Sulforaphane in serum containing medium for 24 h followed by the 72 h treatment of SN38 with or without SFN co-treatment. *Columns*, mean viability as a percentage of control (i.e., cells without chemotherapeutics treatment, 100%) from replicates (n = 6) from three separate experiments; bars, SEM. *** p < 0.001, * p < 0.05 (student's *t*-test).

Surprisingly, we observed that rifampicin did not enhance the resistance of cells to SN38 (figure 4A). These observations suggest that activation of PXR is not required for these effects or that PXR is already activated under these conditions. While neither SN38 nor irinotecan activate PXR (additional file 7), we found that PXR was activated in our assays because of the presence of 10% fetal calf serum, as previously observed in the HepG2 cell line [32]. Accordingly, in presence of serum, CYP3A4 is highly expressed in PXR2 cells, with no additional effect of rifampicin, in contrast to what we observed in absence of serum (figure 4B). Moreover, we found that the pharmacological PXR inhibitor L-sulforaphane (SFN) [33] decreased the percentage of cell survival in PXR2 cells treated with 1 or 5 μM SN38 (15.13% ± 3.99 and 1.72% ± 0.65), compared to cells treated with SN38 alone (31.44% ± 3.31 and 9.32% ± 1.14) (figure 4C). Although we cannot completely exclude off-target effects of SFN, it is likely that inhibition of PXR by SFN contributes toward decreased PXR2 cell resistance.

### Inhibition of PXR expression reverses chemoresistance to SN38

**Figure 5:**
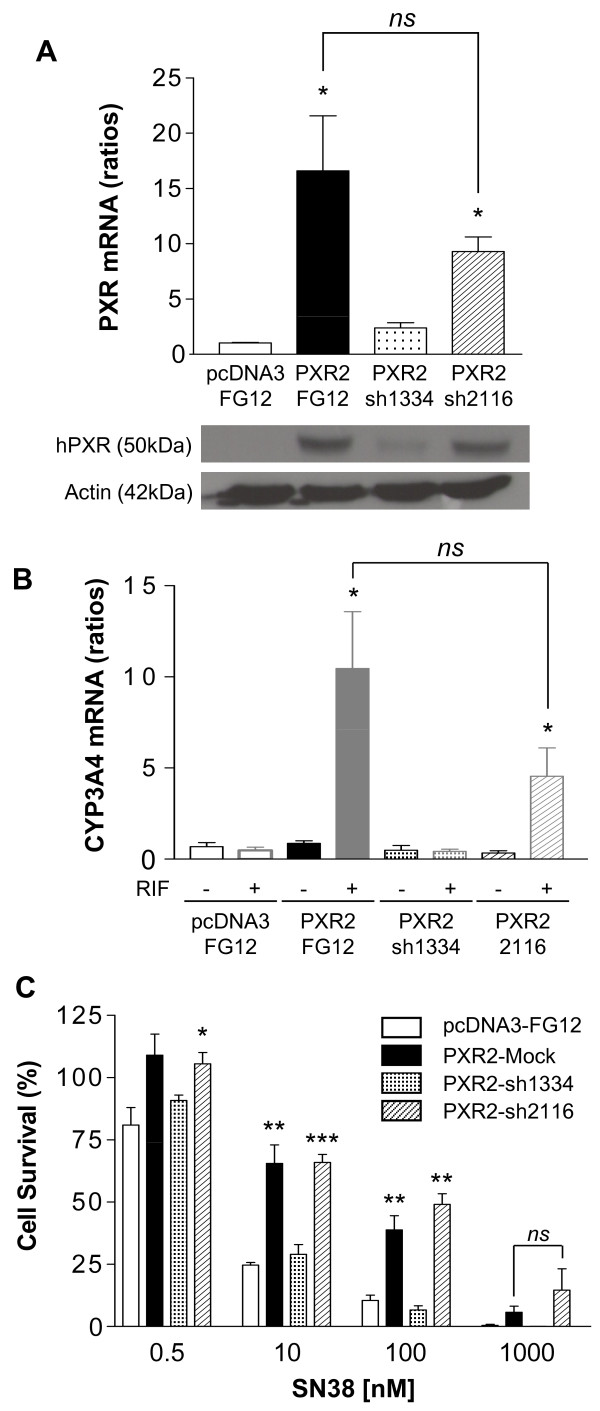
**Reversion of chemoresistance in PXR2 by PXR shRNA**. **A**, quantification of PXR expression by quantitative PCR (top) and western blot (bottom). * p < 0.05 (Mann and Whitney test), PXR expression in PXR2-Mock, PXR2-sh1334 and PXR2-sh2116 cells compared to pcDNA3-Mock cells. **B**, quantification of CYP3A4 mRNA expression after treatment by solvent (DMSO 0.1%) or 10 μM rifampicin for 24 h in serum-free medium. * p < 0.05 (Mann and Whitney test), CYP3A4 expression of cells treated by rifampicin compared to the vehicle treated groups. **C**, cell viability assay on pcDNA3-Mock, PXR2-Mock, PXR2-sh1334 and PXR2-sh2116 toward SN38. *Columns*, mean viability as a percentage of control (i.e., cells without chemotherapeutics treatment, 100%) from replicates (n = 6) from six separate experiments; bars, SEM. Student's *t*-test where performed between CTRL-mock cells to PXR2-Mock, PXR2-sh1334 and PXR2-sh2116: *** p < 0.001, ** p < 0.01, * p < 0.05.

Because SFN is known to affect several other signaling pathways, such as those involving the transcription factors Nrf2 [34] and NF-kappaB [35] that are known to affect cell sensitivity to cytotoxics, we then used a more specific strategy to inhibit PXR expression. For this purpose, LS174T-CTRL or PXR2 cells were transduced with control or shRNA-expressing lentiviral vectors as described in the material and methods section. Although PXR expression was partially decreased in PXR2 cells transduced with control FG12 virus (PXR2-mock) compared to the untransduced PXR2 clone (figure 5A), a stronger decrease was detected in PXR2 cells transduced with shRNA constructs selectively directed against the PXR mRNA. Thus, we observed that PXR2 cells transduced with the sh1334 construct presented a strong decrease of both PXR mRNA and protein levels, while PXR2 transduced with sh2116 displayed a moderate, if any, decrease of PXR expression. Accordingly, induction of CYP3A4 mRNA expression after rifampicin treatment in serum-free medium was observed in PXR2-mock and, to a lesser degree, in PXR2-sh2116 cells, but was lost in PXR2-sh1334 cells (figure 5B). In addition, we carried out neutral red viability assays on these clones (figure 5C), and detected a strong reversion of chemoresistance to SN38 in PXR2-sh1334 cells at 0.1 μM (6.56% ± 1.75 compared to 38.78 ± 5.74 in PXR2-mock), demonstrating that the inhibition of PXR expression by itself is sufficient to enhance chemosensitivity to SN38.

### PXR increases SN38 glucuronidation

Since PXR expression does not affect colon cancer cells proliferation, we hypothesized that SN38 resistance observed in PXR-expressing cells is likely mediated through transcriptional regulation of genes involved in drug metabolism. We first explored several steps of irinotecan metabolism by using pharmacological inhibitors of CYP3A4 (ketoconazole), MDR1 (verapamil) and BCRP (fumitremorgin C). None of these compounds was able to reverse the PXR-dependent chemoresistance (data not shown). We next assessed the metabolic profile of SN38 using a previously described chromatographic detection [27]. Raw data of peak area for intracellular and extracellular SN38 and SN38G allowed us to calculate the metabolic ratios SN38G/SN38.

**Figure 6:**
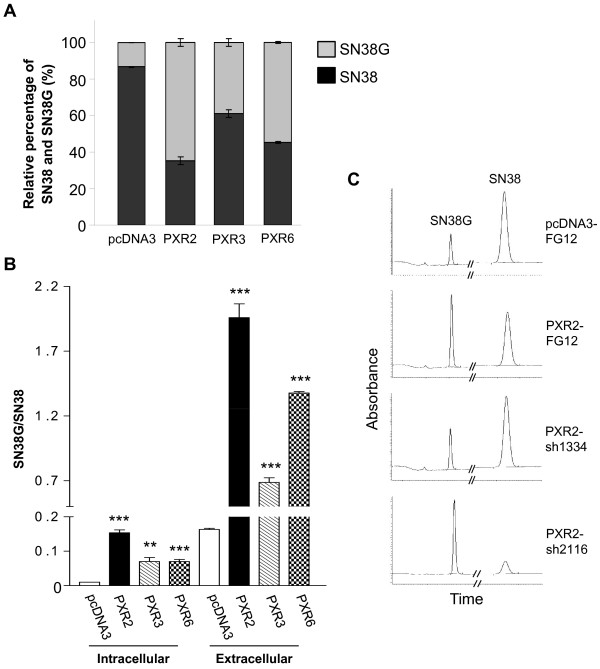
**HPLC quantification of SN38 and SN38G A, Relative percentage of total amounts (intra- and extracellular) of SN38 and SN38G on pcDNA3, PXR2, PXR3 and PXR6 cells after 24 h incubation with 10 μM SN38**. Results were obtained from three separate experiments; bars, SEM. **B**, Intracellular and extracellular SN38G/SN38 ratios on pcDNA3, PXR2, PXR3 and PXR6 cells after 24 h incubation with 10 μM SN38. Results were obtained from three separate experiments; bars, SEM. *** p < 0.001, ** p < 0.01, * p < 0.05 (student's *t*-test) compared to CTRL cells. **C**, representative chromatograms of extracellular medium of pcDNA3-Mock, PXR2-Mock, PXR2-sh1334 and PXR2-sh2116 cells exposed to SN38.

**Figure 7:**
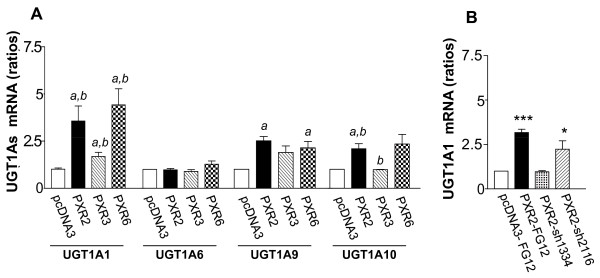
**A, UGT1As mRNA expression levels in pcDNA3, PXR2, PXR3 and PXR6 cells**. Results were obtained from six separate experiments; bars, SEM. ** p < 0.01, * p < 0.05 (student's *t*-test) compared to CTRL cells. **B**, UGT1A1 mRNA expression levels in CTRL-Mock, PXR2-Mock, PXR2-sh1334 and PXR2-sh2116 cells. Results were obtained from four separate experiments; bars, SEM. *** p < 0.001, * p < 0.05 (student's *t*-test) compared to CTRL-Mock cells.

Figure 6A displays relative proportions of total amounts (intra- and extracellular) of SN38 and SN38G. We found a very significant enhancement of SN38 glucuronidation in PXR-transfected cells. As shown in figure 6B, both intracellular and extracellular SN38G/SN38 ratios were significantly increased in cells overexpressing PXR concordant with PXR expression level of clones. Accordingly, PXR inhibition in PXR2-sh1334 cells resulted in a decrease of SN38G levels compared to those found in PXR2-mock or PXR2-sh2116 cells (figure 6C). Because the SN38G/SN38 ratio is a sensitive functional marker of UGT1A1, 6, 9 and 10 enzymes, we measured their relative expression by quantitative RT-PCR. UGT1A1 mRNA expression was found to increase proportionally to the PXR expression levels in PXR-transfected cells (figure 7A). In addition, we also observed a lower, but significant overexpression of both UGT1A9 and UGT1A10 mRNA in those cells, while UGT1A6 mRNA was unaffected. In the same way, we observed a decrease of UGT1A1 mRNA expression in siRNA transfected cells (figure 7B). Taken together, these data strongly suggest that PXR significantly lowers SN38 concentration by increasing SN38 metabolism to its glucuronide conjugate, mainly through induction of UGT1A1.

## Discussion

In this work, we address whether expression of PXR in human colorectal cancer cells could interfere with their sensitivity and metabolism of drugs used in treatment of advanced colorectal cancer. First we showed that hPXR is expressed in both normal and neoplastic human colon tissues with a strong variability in cancer colon tissues. This variability may prove clinically relevant, since a major finding of this study is that expression of PXR in human colorectal cancer cells leads to chemoresistance to the active metabolite of irinotecan, SN38, whereas it did not affect their sensitivity to both 5-fluorouracil and oxaliplatin sensitivities. The opposite effect obtained with pharmacological inactivation of PXR or shRNA-mediated PXR down regulation confirmed the direct involvement of PXR in SN38 chemoresistance. However, in contrast to previous studies showing that PXR affects intrinsic cell survival through the p53 signaling pathway [23] and cell growth [36], we found that PXR induced SN38 chemoresistance in LS174T (p53^*wt*^) as well as in SW480 and SW620 (p53^*mut*^) without affecting their intrinsic proliferation rates. Instead, we observed that PXR expression lowered cellular SN38 concentration while increasing SN38 metabolism to its glucuronide conjugate. Accordingly, we found that several UGT1A isoenzymes were up-regulated in PXR-expressing cells, most notably UGT1A1 which is the key enzyme responsible of the inactivation of SN38 to SN38G.

**Figure 8:**
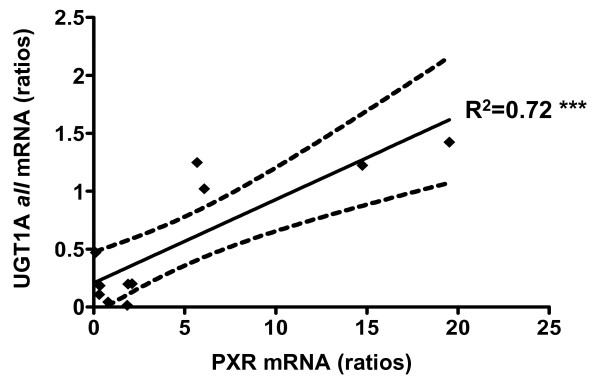
**Correlation of PXR and total UGTs mRNA expression in colon tumors (n = 14)**. Correlation coefficient R^2 ^= 0.72, *** p < 0.001. Doted lines: SEM for a 95% CI.

Since PXR activation (or inhibition) appears to increase (or decrease) SN38 conversion to SN38G, our results highlight the central role of PXR in regulating the cytotoxic threshold of cells to irinotecan-based chemotherapy. Several studies have demonstrated that irinotecan pharmacokinetics and disposition are affected by various compounds now identified as PXR ligands such rifampicin [10], phenobarbital [37], valproic acid and other anticonvulsivant therapies [38,39] or natural products including St. John's wort (*Hypericum perforatum*) [11]. In addition, our data are in accordance with previous studies reporting that intratumoral expression level of UGT1A isoforms may represent a mechanism of intrinsic irinotecan resistance in colon cancer [40,41]. Interestingly a significant (r^2 ^= 0.72, p < 0.001) correlation between PXR and UGT1A1 mRNA levels was found in human colon tumors (figure 8). Taken together, these data suggest that tumoral metabolism is potentially affected by environmental or diet stimuli and this should be taken into account in the prediction of irinotecan disposition in patients. In addition, it is known that diarrhea, a major limiting toxicity of irinotecan, is due to SN38 accumulation in enterocytes [42] and it is conceivable that *in situ *glucuronidation by tumors and adjacent tissues depends on PXR expression levels.

Considering its role as master xenobiotics responsive receptor linking DME genes expression to environment stimuli, we think that differences in PXR expression contribute to the well known intra- and inter-subject variability in irinotecan response, and that they participate in the difficulty to clearly identify factors responsible for pharmacogenetics of irinotecan, the so-called "irinogenetics" [43-45]. Indeed, environmental compounds, nutrition and diet affecting PXR expression and/or activation may mask or attenuate pharmacogenetic associations. Moreover, PXR itself display strong genetic polymorphism with more than 300 reported SNPs in the dbSNPs database, some of which well characterized and inducing differences in both gene expression and ligand recognition [43,44]. PXR expression levels within tumors could be also affected by non-genetic factors such as intra-tumor inflammatory cytokines [46], microRNA 148a [47] and methylation status of its exon 3 [48]. In this context, discriminating the roles of genetic influences from environmental effects in drug response, recently coined "pharmacoecology" [49], will be even harder as expected. Thus, it will be of interest to evaluate the relative importance of these genetic and non-genetic factors in patient response toward irinotecan-based chemotherapy.

## Conclusion

In view of the present findings, clinical studies are now needed to evaluate the potential interest of PXR in personalized medicine. Indeed, PXR expression and/or activation level could help physicians in the choice of appropriate chemotherapy regimen for colorectal cancer patients, since therapeutic alternatives to irinotecan already exist (*i.e*. platinum salt or targeted therapy). Finally, PXR down-regulation could be considered as a novel therapeutic approach to circumvent chemoresistance to chemotherapy.

## Competing interests

The authors declare that they have no competing interests.

## Authors' contributions

CR and JMP designed and carried out most of the experiments and wrote the initial drafts of the manuscript. GL carried the cell viability assays studies on shRNA clones. CB (Breuker) carried out the western blots analysis of PXR. JK and BL provided valuable help in RT-QPCR experiments. CB (Bonnans) carried out immunohistochemistry of PXR. SP supervised HPLC experiments. DJ and FH made original observations leading to this work and contributed to the critical revision of the manuscript. JPB and SL provided valuable reagents and devices and contributed to data interpretation. AE made first assumptions and contributed to the conception and design of the entire study and the final editing of the manuscript. All authors read and approved the final manuscript.

## Supplementary Material

Additional file 1 **Sequences of shRNA cassettes used for the inhibition of PXR expression**. The shRNA-expressing vectors were constructed by cloning shRNA expression cassettes into FG12 lentiviral vector. Click here for file

Additional file 2 **Primers sequences used for the quantification of PXR and its target genes by qPCR**. mRNAs expression was evaluated by RT-quantitative PCR using a LightCycler 480 real-time PCR system and gene-specific primers, β-actin was used as reference gene. Click here for file

Additional file 3 **Topoisomerase I activity in LS174T pcDNA3 and PXR-transfected cells**. Topoisomerase I activity was assessed by using a kit from TopoGen based on the ability of nuclear extracts to yield relaxed plasmid from supercoiled plasmid substrate DNA. Nuclear extracts from LS174T pcDNA3 and PXR-transfected cells were incubated for 15, 30 and 60 minutes with a supercoiled DNA marker subsequently subjected to agarose electrophoresis. Click here for file

Additional file 4 **Carboxylesterases mRNA quantification**. Carboxylesterases (CES1 and CES2) expression level in LS174T pcDNA3 transfected-cells, stable clone PXR2 and a pool of cDNA extracted from liver biopsies. Results were obtained from six separate experiments; bars, SEM. Click here for file

Additional file 5 **Characterization of SW480 and SW620 PXR-transfected cells**. **A**, **C**, PXR expression level in control and stable clones SW480-PXR1, SW620-1L and SW620-1H (*U*, undetectable PXR expression level). LS174T CTRL cells were taken as a calibrator for quantitative PCR. *** p < 0.001, PXR expression of stable clones compared to SW620 or SW480 control cells, assayed by Student's *t*-test. **B**, **D**, Increased chemoresistance in PXR overexpressing cells to SN38. For neutral red assays, cells were treated for 72 h by increasing concentrations of SN38. *Columns*, mean viability as a percentage of control (i.e., cells without chemotherapeutics treatment, 100%) from replicates (n = 6) from three separate experiments; bars, SEM. * p < 0.05, viability percentages of PXR-expressing cells compared to control cells (Student's *t*-test). Click here for file

Additional file 6 **Cell viability assays of LS174T control and PXR expression cells, PXR2 and PXR6, to 5-FU and oxaliplatine**. For neutral red assays, cells were treated for 72 h by increasing concentrations of 5-FU (**A**) or oxaliplatine (**B**). *Columns*, mean viability as a percentage of control (i.e., cells without chemotherapeutics treatment, 100%) from replicates (n = 6) from three separate experiments; bars, SEM. Click here for file

Additional file 7 **CYP3A4 mRNA quantification after drug treatment**. CYP3A4 mRNA expression levels in pcDNA3, PXR2, PXR3 and PXR6 cells after treatment with irinotecan or SN38. Results were obtained from six separate experiments; bars, SEM. Click here for file
