# Supplementary material for: Pregnane × Receptor (PXR) expression in colorectal cancer cells restricts irinotecan chemosensitivity through enhanced SN-38 glucuronidation
Source: Mol Cancer. 2010 Mar 2;9:46. doi: 10.1186/1476-4598-9-46 (PMC2838814; doi:10.1186/1476-4598-9-46)
Supplement: Additional file 1 — Sequences of shRNA cassettes used for the inhibition of PXR expression. The shRNA-expressing vectors were constructed by cloning shRNA expression cassettes into FG12 lentiviral vector. [file 1476-4598-9-46-S1.PDF]

**shRNA name**	**Sequence (5’-3’)**
PXR-1334	ACCGGAGCAATTCGCCATTACTTTCAAGAGAAGTAATGGCGAATTGCTCCTTTTTC
	TCGAGAAAAAGGAGCAATTCGCCATTACTTCTCTTGAAAGTAATGGCGAATTGCTC
PXR-2116	ACCGTCTCTGCATCCATTTGAATTCAAGAGATTCAAATGGATGCAGAGACTTTTTC
	TCGAGAAAAAGTCTCTGCATCCATTTGAATCTCTTGAATTCAAATGGATGCAGAGA
