# Supplementary material for: Pregnane × Receptor (PXR) expression in colorectal cancer cells restricts irinotecan chemosensitivity through enhanced SN-38 glucuronidation
Source: Mol Cancer. 2010 Mar 2;9:46. doi: 10.1186/1476-4598-9-46 (PMC2838814; doi:10.1186/1476-4598-9-46)
Supplement: Additional file 2 — Primers sequences used for the quantification of PXR and its target genes by qPCR. mRNAs expression was evaluated by RT-quantitative PCR using a LightCycler 480 real-time PCR system and gene-specific primers, β-actin was used as reference gene. [file 1476-4598-9-46-S2.PDF]

**Primer name**	**Sequence (5’-3’)**	**Experimental Tm (°C)**
PXR forward	GGCATGAAGAAGGAGATGAT	60
PXR reverse	TGGGAGAAGGTAGTGTCAAA	
CYP3A4 forward	TATTCTGTCTTCACAAACCG	60
CYP3A4 reverse	TTTCTCACCAACACATCTCC	
MDR1 forward	GGTGCTGGTTGCTGCTTACA	65
MDR1 reverse	TGGCCAAAATCACAAGGGT	
CES1 forward	CTGTGATTGATGGGATGCTG	65
CES1 reverse	TGCAAACAAGGGGATAGGAC	
CES2 forward	GTAGCACATTTTCAGTGTTCC	60
CES2 reverse	GTAGTTGCCCCCAAAGAA	
UGT1A1 forward	AAATCCACTATCCCAGGAAT	60
UGT1A1 reverse	AGTATCGTGTTGTTCGCAAG	
UGT1A6 forward	CTTTTCACAGACCCAGCCTTAC	63
UGT1A6 reverse	TATCCACATCTCTCTTGAGGACAG	
UGT1A9 forward	GAGGAACATTTATTATGCCACCG	63
UGT1A9 reverse	GCAACAACCAAATTGATGTGTG	
UGT1A10 forward	CCTCTTTCCTATGTCCCCAATGA	63
UGT1A10 reverse	CAACAACCAAATTGATGTGTG	
UGT1A*all* forward	CGAATCTTGCGAACAACACGAT	63
UGT1A*all* reverse	TTCAGGGTCACTCCAGCTCC	
β-Actin forward	AGCACGGCATCGTCACCAACT	65
β-Actin reverse	TGGCTGGGGTGTTGAAGGTCT	
