# Supplementary figures and images for: Pregnane × Receptor (PXR) expression in colorectal cancer cells restricts irinotecan chemosensitivity through enhanced SN-38 glucuronidation
Source: Mol Cancer. 2010 Mar 2;9:46. doi: 10.1186/1476-4598-9-46 (PMC2838814; doi:10.1186/1476-4598-9-46)

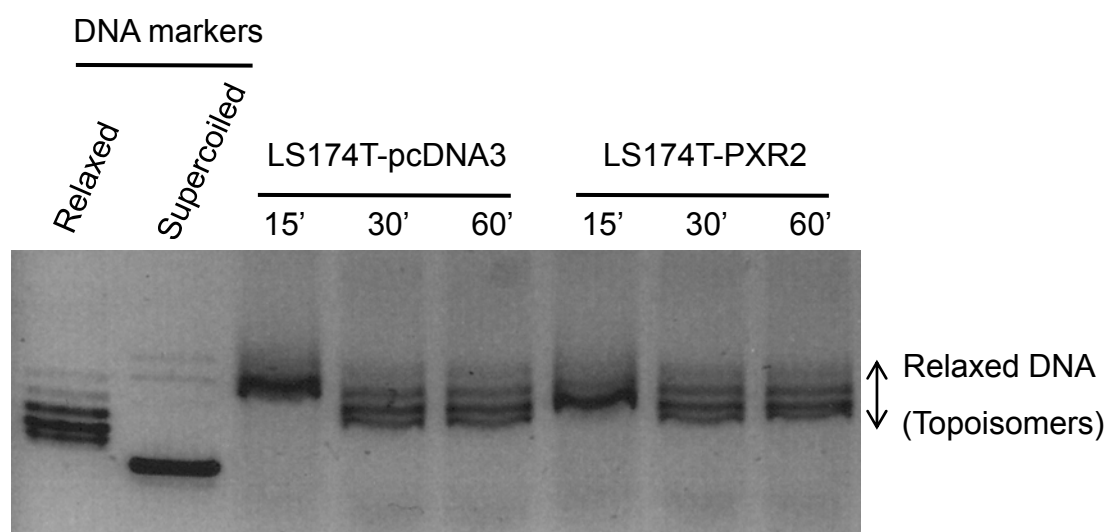

Supplement: Additional file 3 — Topoisomerase I activity in LS174T pcDNA3 and PXR-transfected cells. Topoisomerase I activity was assessed by using a kit from TopoGen based on the ability of nuclear extracts to yield relaxed plasmid from supercoiled plasmid substrate DNA. Nuclear extracts from LS174T pcDNA3 and PXR-transfected cells were incubated for 15, 30 and 60 minutes with a supercoiled DNA marker subsequently subjected to agarose electrophoresis. [file 1476-4598-9-46-S3.PDF]

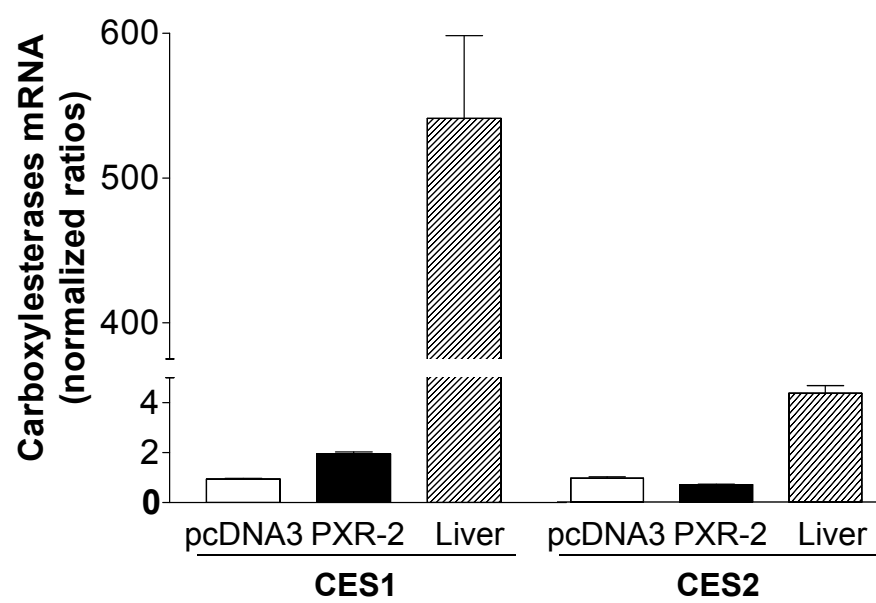

Supplement: Additional file 4 — Carboxylesterases mRNA quantification. Carboxylesterases (CES1 and CES2) expression level in LS174T pcDNA3 transfected-cells, stable clone PXR2 and a pool of cDNA extracted from liver biopsies. Results were obtained from six separate experiments; bars, SEM. [file 1476-4598-9-46-S4.PDF]

**A**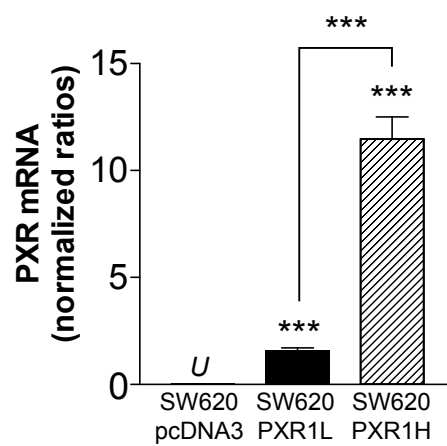**B**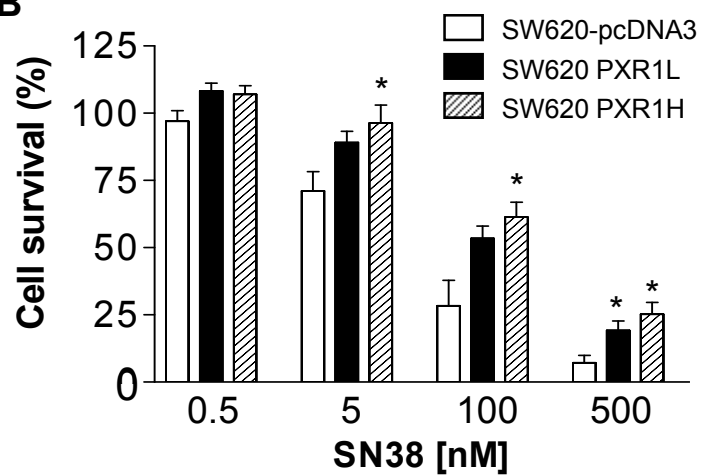**C**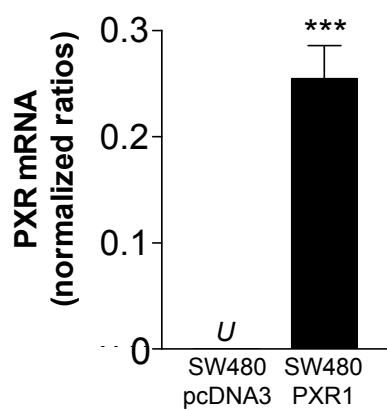**D**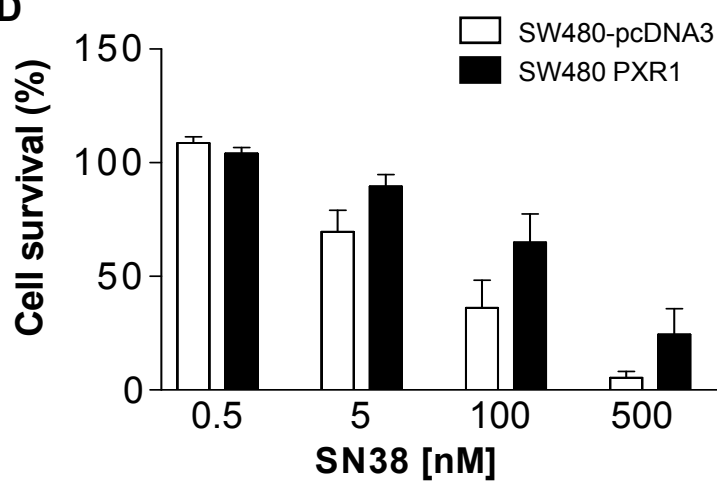

Supplement: Additional file 5 — Characterization of SW480 and SW620 PXR-transfected cells. A, C, PXR expression level in control and stable clones SW480-PXR1, SW620-1L and SW620-1H (U, undetectable PXR expression level). LS174T CTRL cells were taken as a calibrator for quantitative PCR. *** p < 0.001, PXR expression of stable clones compared to SW620 or SW480 control cells, assayed by Student's t-test. B, D, Increased chemoresistance in PXR overexpressing cells to SN38. For neutral red assays, cells were treated for 72 h by increasing concentrations of SN38. Columns, mean viability as a percentage of control (i.e., cells without chemotherapeutics treatment, 100%) from replicates (n = 6) from three separate experiments; bars, SEM. * p < 0.05, viability percentages of PXR-expressing cells compared to control cells (Student's t-test). [file 1476-4598-9-46-S5.PDF]

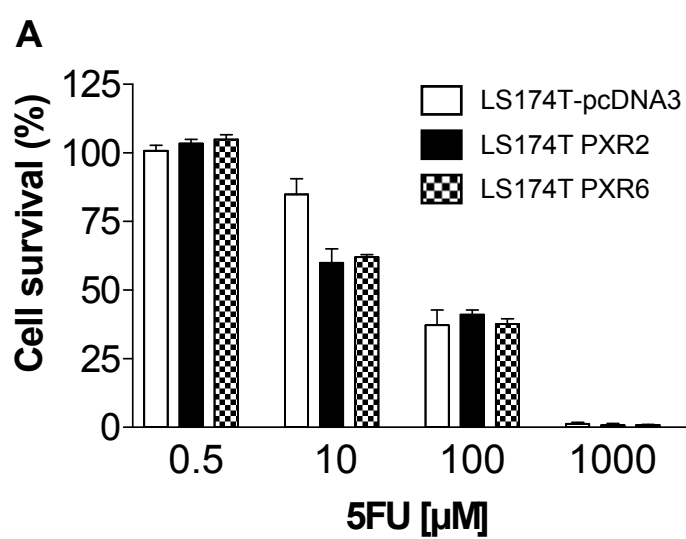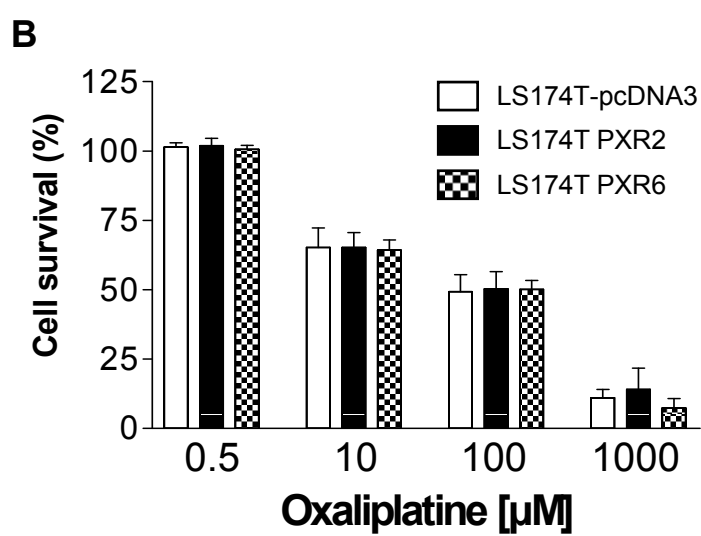

Supplement: Additional file 6 — Cell viability assays of LS174T control and PXR expression cells, PXR2 and PXR6, to 5-FU and oxaliplatine. For neutral red assays, cells were treated for 72 h by increasing concentrations of 5-FU (A) or oxaliplatine (B). Columns, mean viability as a percentage of control (i.e., cells without chemotherapeutics treatment, 100%) from replicates (n = 6) from three separate experiments; bars, SEM. [file 1476-4598-9-46-S6.PDF]

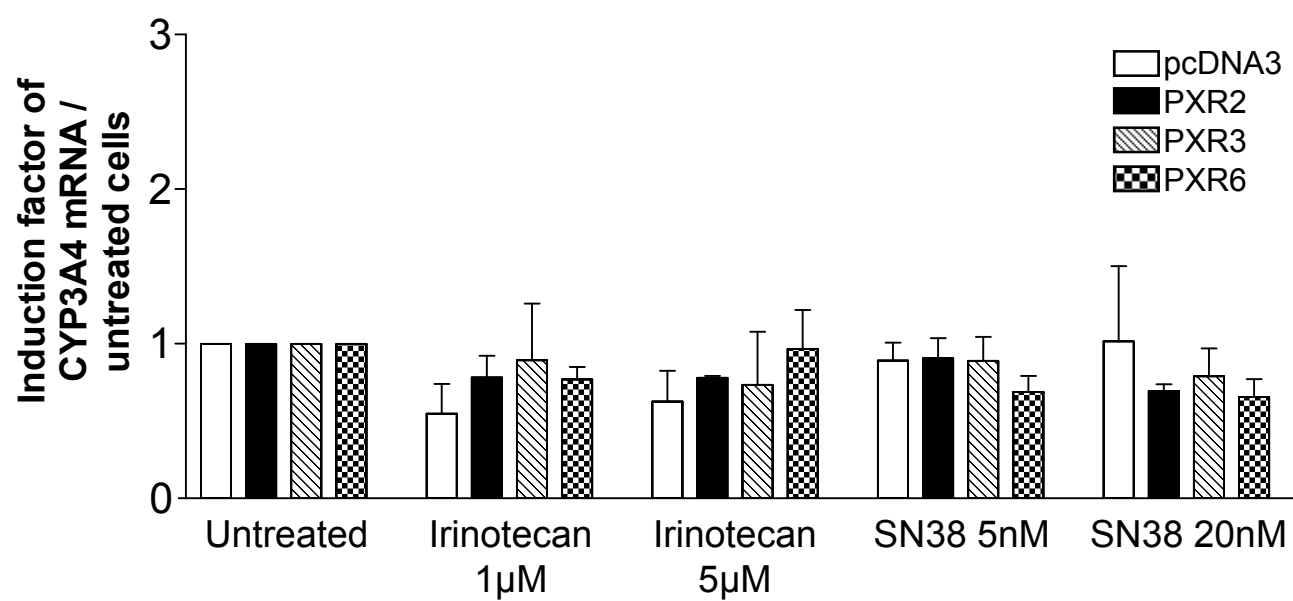

Supplement: Additional file 7 — CYP3A4 mRNA quantification after drug treatment. CYP3A4 mRNA expression levels in pcDNA3, PXR2, PXR3 and PXR6 cells after treatment with irinotecan or SN38. Results were obtained from six separate experiments; bars, SEM. [file 1476-4598-9-46-S7.PDF]
